# Malaria Vector Surveillance and Control in an Elimination Setting in South Africa

**DOI:** 10.3390/tropicalmed7110391

**Published:** 2022-11-21

**Authors:** Basil D. Brooke

**Affiliations:** 1 Centre for Emerging Zoonotic and Parasitic Diseases, National Institute for Communicable Diseases/NHLS, Johannesburg 2131, South Africa; basilb@nicd.ac.za; 2 Wits Research Institute for Malaria, Faculty of Health Sciences, University of the Witwatersrand, Johannesburg 2050, South Africa

**Keywords:** *Anopheles*, vector control, low-incidence settings, risk

## Abstract

South Africa’s malaria elimination plans are aligned to the World Health Organization’s aim for a malaria-free world and include specific objectives within a specified time frame. These are proving difficult to achieve owing to the sporadic nature of locally acquired malaria in some affected districts, while other districts that were endemic for the disease are either malaria-free or very close to that goal. The WHO also specifies that continued measures to prevent the re-establishment of transmission are required in areas where elimination has been achieved. These measures include routine malaria vector surveillance in endemic districts that are free of malaria to assess receptivity and risk of reintroduction, which may prove difficult to justify in the face of competing public health priorities and limited resources. These issues are discussed here within the framework of vector surveillance and control and include recommendations on how they can be addressed going forward.

## 1. Malaria and Plans for Elimination in South Africa

The World Health Organization defines malaria elimination as “the interruption of local transmission of a specified malaria parasite species in a defined geographical area as a result of deliberate activities” [1]. Following decades of successful control, malaria incidence in South Africa is now low enough to scale interventions toward elimination.

**Figure 1:**
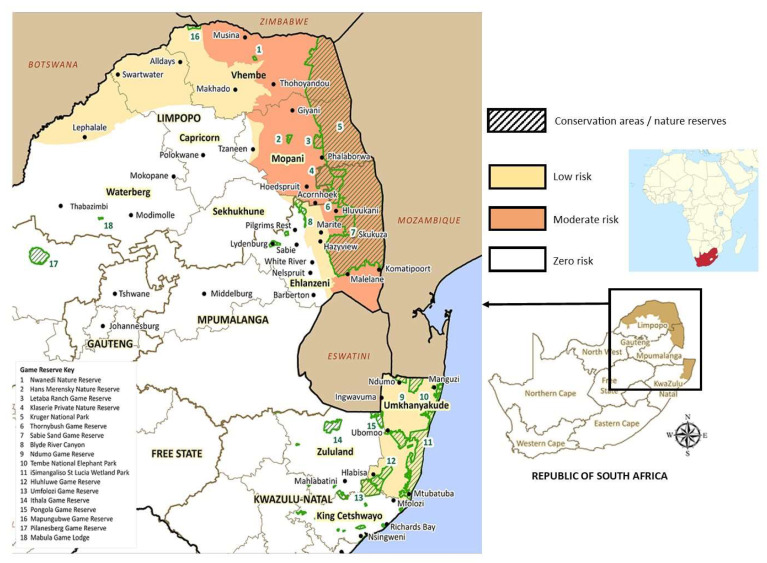
Malaria risk map for South Africa by endemic province and district/municipality. Adapted from and courtesy of the National Department of Health and Medical Research Council, South Africa (https://www.health.gov.za/malaria/, accessed 10 November 2022).

Most malaria cases recorded in South Africa are imported, with low-level locally acquired malaria restricted to a comparatively small number of districts in the three endemic provinces: KwaZulu-Natal, Mpumalanga and Limpopo (Figure 1). These districts collectively host 20 malaria-endemic municipalities. The incidence of locally acquired malaria varies between districts. For example, in KwaZulu-Natal Province, the UMkhanyakude District records 20–100 local cases annually, Zululand District records 2–20 local cases annually and King Cetshwayo District records 3–15 local cases annually. South Africa’s national malaria incidence is currently very low with 0.49/1000 of the population at risk overall, and locally acquired cases account for 0.11/1000 of the population. South Africa is, therefore, a frontline country in terms of the drive toward malaria elimination at the sub-national and national levels [2,3,4,5,6].

Malaria in South Africa is primarily caused by *Plasmodium falciparum* (>97%) with very small contributions from *P. ovale*, *P. malariae* and *P. vivax* [5]. Point-of-care diagnosis is typically based on the use of rapid diagnostic tests (RDTs). Referrals and complicated cases are confirmed by microscopy and/or polymerase chain reaction (PCR) assay at specialized laboratories. Although RDT stockouts do occasionally occur, supplies are generally well-managed. Microscopy proficiency schemes and quality assurance assessments are routinely coordinated by partner institutions [7].

The malaria incidence in South Africa is seasonal and tends to peak in late summer after significant rainfall [5,6]. Localized outbreaks and broader epidemics can, however, occur. These events are generally linked to unusual weather patterns such as heavy rainfall in particular and high humidity, and can also be caused by large-scale migrations of people across borders (thereby, the mass importation of malaria parasites via infected people). They can also be caused by the sub-optimal implementation of control interventions. The epidemic that occurred in South Africa during the period from 1996 to 2000 was primarily attributed to anti-malarial drug resistance in *Plasmodium* parasite populations and insecticide resistance in *Anopheles* mosquito vector populations, which led to programmatic failure despite the implementation of interventions [8,9].

South Africa’s malaria elimination agenda for the period from 2019 to 2023 is based on five broad strategic objectives [10] built on the long experience of malaria control in South Africa [11]:

1. Provision of effective management, leadership and coordination;
2. Provision of sustainable and strengthened surveillance systems ensuring that 100% of cases are reported to the Malaria Information System (MIS);
3. Community outreach and the provision of pertinent malaria information;
4. Measures to ensure that all populations at risk receive at least 95% coverage with key vector suppression strategies and interventions;
5. Universal access to diagnosis and treatment in endemic and non-endemic areas according to national guidelines.

Vector control (objective 4) in the endemic provinces includes either blanket or targeted indoor residual spraying (IRS) of specially formulated insecticides [12] depending on recent historical incidence and the estimated levels of risk per endemic district [13,14]. Larval source management based on the WHO’s ‘few, fixed and findable’ approach and winter larviciding of known permanent *Anopheles* breeding sites complement the IRS campaigns [10]. Cross-border collaborations with neighboring countries coordinated through the Elimination 8 and MOSASWA initiatives actively promote and support vector control and other interventions in border districts [2,4]. Vector surveillance is conducted by provincial entomology teams with support from partner institutions. Recommended key indicators are aligned with the WHO guidelines and include vector species assemblage, insecticide susceptibility, breeding, feeding and resting behaviors [1,10,11]. This information is yet to be realized for many affected districts.

## 2. Sporadic Malaria in Low Incidence Settings

Despite annual vector control interventions, locally acquired malaria persists in South Africa. It is often characterized by an unusual epidemiological profile in low-incidence settings whereby cases are sporadic as opposed to ongoing or recurrent transmission characteristics of endemicity in higher incidence settings [15], such as the Vhembe District of Limpopo Province—a hotspot for local malaria transmission [16] (Figure 1). This sporadic incidence pattern is based on the locations of index houses which appear to be randomly distributed within the context of malaria transmission, i.e., they are often not in the immediate vicinity of one another and there are no obvious epidemiological links between cases from these dwellings. Such a sporadic incidence suggests that index houses are not necessarily the actual sources of infection, and that local social customs, community interactions and events or gatherings at specific localities might be especially important in terms of transmission. Such gatherings could include sports events, religious services, community meetings, celebrations, festivals and social hubs (including restaurants and taverns). This possibility has important implications for malaria control and elimination because it implies a need for control interventions at such places and provides an opportunity for community-level active case detection.

A significant proportion of locally acquired malaria cases in South Africa are now classified as secondarily imported cases. These occur when local vector mosquitoes acquire malaria infections from infected (often asymptomatic) people who are migrants from or have recently travelled to a malaria-endemic area such as a neighboring country. This can lead to sporadic clusters of local malaria when infected mosquitoes subsequently bite local residents. An example is sporadic clusters of local cases on sugarcane farms in Zululand District during the planting and harvesting seasons.

The importation of infective mosquitoes from neighboring countries or other endemic areas within South Africa is also a possible explanation for at least some of the sporadic locally acquired cases. Odyssean malaria (also known as airport or taxi malaria) occurs via the inadvertent transfer of one or several infective mosquitoes via land or air transport. Such mosquitoes can alight from vehicles at some point during their journey from where they bite and infect one or more local residents who happen to be in close proximity to where the infective mosquito/es escaped (typically within a few hundred meters of the escape point). Although it is not possible to distinguish between odyssean and local malaria in endemic areas, a proxy for the occurrence of this category of malaria comes from its occurrence in non-endemic provinces (i.e., no malaria transmission) within South Africa, especially Gauteng Province. Between two and ten odyssean malaria cases are recorded in Gauteng Province annually, their classification being based on affected patients having no travel history to a malaria-affected area in the month preceding the onset of symptoms [17]. If odyssean malaria occurs in Gauteng Province and occasionally in other non-endemic provinces, it is reasonable to assume that some local cases in the endemic provinces are also odyssean, which would explain the sporadic nature of their occurrence.

The sporadic nature of local malaria incidences in many of the affected low-incidence districts is problematic for malaria elimination because it is difficult to plan targeted vector control interventions without having a reliable predictor of where future cases are most likely to occur. This can be addressed by the foci-clearing protocols that are based on a set of reactive measures when one or more cases occur. These measures include active case detection and treatment of any infected people resident in or in the vicinity of the index house, vector control in index houses and their immediate neighbors, larval source management in the vicinity of the index house and vector surveillance. It is, therefore, the author’s opinion that vector control measures, suitably implemented, can enable malaria elimination by reducing incidences to very low levels, but the actual elimination by districts then depends on parasite clearance by active and passive case detection.

## 3. Assessing Risk and Receptivity in Endemic Areas Cleared of Malaria

Some districts and municipalities in South Africa’s malaria-endemic provinces (such as King Cetshwayo District in KwaZulu-Natal Province and Waterberg District Municipality in Limpopo Province) are almost malaria-free in terms of local transmission. This does not, however, mean that they are safe from the reintroduction of malaria by the importation of *Plasmodium* parasites via the movements of infected people. What matters is how receptive these areas are to malaria transmission [18].

Malaria can only be acquired through the bite of an infective *Anopheles* mosquito. The only possible and extremely rare exception to this rule is the acquisition of malaria by blood transfusion. Assessing the risk of malaria in any given area or region is therefore based on the occurrence of *Anopheles* vector populations. These are generally classified as either major or secondary vectors via a loosely defined classification system based on their measured or inferred contributions to malaria transmission. Five *Anopheles* species are implicated as malaria vectors within South Africa’s borders [19,20,21]. Two of these are the major vectors *Anopheles funestus sensu stricto* and *An. arabiensis*. These two species tend to associate closely with human communities (especially *An. funestus*) from whom they acquire most of their blood meals, and are, therefore, responsible for the bulk of locally acquired malaria cases. Secondary vectors include *An. vaneedeni*, *An. parensis* and *An. merus*. They are listed as secondary vector species either because they interact with human communities to a lesser extent than the major vectors, because they are rarer, or because they are less susceptible to *Plasmodium* infections and are, therefore, comparatively inefficient vectors. Secondary vectors can, however, play prominent roles in malaria transmission and should not be dismissed during vector control operational research and planning [22].

Assessing risk and receptivity in a malaria-free area that was previously endemic for the disease involves regular surveillance for Anopheles mosquitoes. This is a complex task requiring skilled entomologists who are able to employ a range of methods designed to catch adult and aquatic-stage Anopheles mosquitoes [23]. Processing these collections in order to collect essential surveillance indicators requires detailed planning, sophisticated laboratory infrastructure and a highly specialized skill set amongst the personnel dedicated to this task. The essential indicators include species identification leading to the compilation of an *Anopheles* species assemblage for the area in question, insecticide susceptibility profiles of each vector population, and information concerning their feeding (indoor or outdoor and peak biting times), resting (indoor or outdoor) and breeding characteristics (characteristics of water bodies that they choose for egg deposition). This information enables an assessment of the risk of malaria reintroduction, and can be used for planning vector control operations—i.e., appropriate methods and insecticide choice—should reintroduction occur.

## 4. Conclusions

Importantly, here there is the recognition of the need for two critical interventions: ongoing surveillance and control including outbreak response. Intensive malaria parasite and vector surveillance pre- and post-elimination will need to be maintained [23,24]. Many of South Africa’s neighboring countries, especially Mozambique and Zimbabwe, are highly endemic for malaria and so the risks associated with importation and reintroduction are high. Commitment to ongoing surveillance and control in the face of zero local cases and competing public health interests will need to be maintained. Malaria elimination and the maintenance of that status will require strong advocacy from all stakeholders, and continued readiness of the provincial control programs to respond to outbreaks using local contingency plans, the foci-clearing protocols and vector surveillance information.

## 5. Recommendations

- Low-incidence districts earmarked for malaria elimination should ensure the full implementation of proactive or reactive vector control methods year on year, even after malaria elimination is achieved.
- Low incidence districts earmarked for malaria elimination should plan, implement and maintain active and passive case detection, especially amongst migrant and immigrant communities.
- Assessments of malaria risk and receptivity in malaria-free districts in endemic provinces should be routinely conducted by provincial entomological surveillance officers on an annual basis.
- Annual vector control plans at the local (district/municipality) level should be based on *Anopheles* species assemblages and insecticide susceptibility profiles of known vector populations.
- Additional vector control methods including dual active ingredient insecticide-treated bed net distribution, community outreach in terms of personal protection methods, housing design and screening, and environmental management (such as drainage of non-utilized water bodies used by mosquitoes for breeding) should be considered pre- and post-malaria elimination at the local level.
- Political commitment and the resources required for ongoing surveillance and control operations will need to be sourced year on year from national and provincial governments. This will require a multi-stakeholder approach coordinated by the NDoH Malaria Directorate. Important government department stakeholders include Agriculture, Finance and Environment.
- Cross-border initiatives need to be maintained, sustained and strengthened as needed, especially in terms of vector and parasite surveillance at border crossings/posts.
